# Supplementary material for: Zero-inflated Poisson regression analysis of factors associated with under-five mortality in Ethiopia using 2019 Ethiopian mini demographic and health survey data
Source: PLoS One. 2023 Nov 10;18(11):e0291426. doi: 10.1371/journal.pone.0291426 (PMC10637676; doi:10.1371/journal.pone.0291426)
Supplement: Attachment — Submitted filename: Response to Reviewers.docx [file pone.0291426.s002.docx]

**Authors’ response to Editor and reviewers**

Dear Editor,

Thank you so much for allowing resubmission of our manuscript with an opportunity to address the reviewers’ comments that could help to enhance the quality of the manuscript.

We would also like to express our gratitude to the anonymous reviewer for carefully reviewing the manuscript and for many thoughtful comments, which can enhance the readability and quality of this manuscript.

We are uploading (i) our point-by-point response to the Editor’s and reviewer’s comments, and (ii) updated manuscript with changes being highlighted by red colour.

Best regards,

Alemayehu Siffir Argawu, the corresponding author of the manuscript

**Author’s response for Academic editor comments**

1. Please ensure that your manuscript meets PLOS ONE's style requirements, including those for file naming.

Authors’ Response: Thank you so much for the comment. The manuscript has been updated as per the PLOS ONE style requirements.

2. We note that you have indicated that data from this study are available upon request. PLOS only allows data to be available upon request if there are legal or ethical restrictions on sharing data publicly. For more information on unacceptable data access restrictions, please see http://journals.plos.org/plosone/s/data-availability#loc-unacceptable-data-access-restrictions.

In your revised cover letter, please address the following prompts:

a) If there are ethical or legal restrictions on sharing a de-identified data set, please explain them in detail (e.g., data contain potentially sensitive information, data are owned by a third-party organization, etc.) and who has imposed them (e.g., an ethics committee). Please also provide contact information for a data access committee, ethics committee, or other institutional body to which data requests may be sent.

Authors’ response: The data we drawn, for the analysis of this study, is publically open secondary data which is available on the DHS website (http:// dhsprogram.com). We got permission from Demographic and Health Surveys (DHS) Program and downloaded data. As this data is publicly available and has no personal identifiers, Ethical approval was not necessary.

b) If there are no restrictions, please upload the minimal anonymized data set necessary to replicate your study findings as either Supporting Information files or to a stable, public repository and provide us with the relevant URLs, DOIs, or accession numbers. For a list of acceptable repositories, please see http://journals.plos.org/plosone/s/data-availability#loc-recommended-repositories.

We will update your Data Availability statement on your behalf to reflect the information you provide.

Authors’ Response: All data used for the final analysis for this study are available from corresponding author. We modified the statement in the submission system and uploaded the data.

3. We note that you have stated that you will provide repository information for your data at acceptance. Should your manuscript be accepted for publication, we will hold it until you provide the relevant accession numbers or DOIs necessary to access your data. If you wish to make changes to your Data Availability statement, please describe these changes in your cover letter and we will update your Data Availability statement to reflect the information you provide.

Authors’ Response: We modified it in the Editorial system.

4. PLOS requires an ORCID iD for the corresponding author in Editorial Manager on papers submitted after December 6th, 2016. Please ensure that you have an ORCID iD and that it is validated in Editorial Manager. To do this, go to ‘Update my Information’ (in the upper left-hand corner of the main menu), and click on the Fetch/Validate link next to the ORCID field. This will take you to the ORCID site and allow you to create a new iD or authenticate a pre-existing iD in Editorial Manager. Please see the following video for instructions on linking an ORCID iD to your Editorial Manager account: <https://www.youtube.com/watch?v=_xcclfuvtxQ>.

Authors’ response: The ORCID iD is provided now.

**Authors’ response to Reviewer**

Dear Reviewer, thank you so much for your very constructive and crucial comments that help for the improvement of the manuscript.

1. Abstract: The result written in the abstract section is needed to re-write.

Authors’ Response: Thank you, dear reviewer, very much for comment. We have rewritten the result section.

2. Variable in study not been define clearly.

Authors’ Response: Thank you, dear reviewer, very much for the comment. Now, the study variables, **response variable** and **independent variables** are clearly and separately put.

3. I am sceptical about the method used in the study; survival regression can be used in spite of Zero-inflated Poisson regression.

Authors’ Response: Thank you so much dear reviewer. In this study, the response/outcome variable was the number of children died before celebrating fifth birthday per woman in her lifetime. This **‘the number of children died before the age of 5 years’** can be zero if none of the children of mother died before the age of 5 years i.e, if mother didn’t lose under-five children in her lifetime, the number of the children died before the age of 5 years is considered zero. It can be 1 if a woman lost one child before turning age 5. It can also be 2, 3,…. As it is clearly known the response variable is count i.e, it can take on 0 or 1 or 2 or,…. In this study, the number of children died before age of 5 years ranged from 0 to 7. The appropriate models to analyse such count response data are the count regression models. For the excess zero outcome of the count data, Zero-inflated Poisson regression is appropriate. Based on the model comparison criteria, Zero-Inflated Poisson (ZIP) regression model was found to be the best model to fit the data. And, based on your comment, we modified the title as Zero-Inflated Poisson (ZIP) regression analysis of factors associated under-five mortality as the ZIP regression model best fitted the data compared to other count regression models used in the study.

4. Justifications of the methodology used has to be added.

Authors’ Response: Thank you, dear reviewer, so much. We added justification of the methodology used in the manuscript under section Statistical Data Analysis Methods

5. What is newness in the study? and how it different from the existing studies form the same dataset.

Authors’ Response: Thank you, dear reviewer, for the question. Many of the studies previously done were analysed the data using logistic regression and survival regression analysis. But, this study was done analysing the data using the count regression analysis models. As the under-five mortality data experiences excess zero (0), the Zero-Inflated Poisson (ZIP) regression model was used. Moreover, this study included some additional important variables, such as type of cooking fuel, time to get source of water, age of household head, relationship to household head, number of under-five children, and number of children at home, which were not included in most of the previously studies from the same dataset. And, three new factors of under-five mortality, such as mothers’ time to get source of drinking water, household heads ages, and number of children at home, were found to be significant factors in this study as compared with previously conducted studies in Ethiopia.
